# Change in Androgenic Status and Cardiometabolic Profile of Middle-Aged Women with Polycystic Ovary Syndrome

**DOI:** 10.3390/jcm12165226

**Published:** 2023-08-11

**Authors:** Kim van der Ham, Maria P. H. Koster, Birgitta K. Velthuis, Ricardo P. J. Budde, Bart C. J. M. Fauser, Joop S. E. Laven, Yvonne V. Louwers

**Affiliations:** 1 Division of Reproductive Endocrinology and Infertility, Department of Obstetrics and Gynecology, Erasmus University Medical Center, Dr. Molewaterplein 40, 3015 GD Rotterdam, The Netherlandsj.laven@erasmusmc.nl (J.S.E.L.);; 2 Department of Radiology, University Medical Center Utrecht, University of Utrecht, Heidelberglaan 100, 3584 CX Utrecht, The Netherlands; 3 Department of Radiology and Nuclear Medicine, Erasmus University Medical Center, Dr. Molewaterplein 40, 3015 GD Rotterdam, The Netherlands; 4 Department of Reproductive Medicine & Gynecology, University Medical Center Utrecht, University of Utrecht, Heidelberglaan 100, 3584 CX Utrecht, The Netherlands

**Keywords:** PCOS, hyperandrogenism, cardiometabolic profile, post-reproductive years

## Abstract

Understanding the cardiovascular disease (CVD) risk for women with polycystic ovary syndrome (PCOS) at reproductive age is crucial. To investigate this, we compared the cardiometabolic profiles of different PCOS groups over a median interval of 15.8 years. The study focused on three groups: (1) women with PCOS who were hyperandrogenic at both initial and follow-up screening (HA-HA), (2) those who transitioned from hyperandrogenic to normoandrogenic (HA-NA), and (3) those who remained normoandrogenic (NA-NA). At initial and follow-up screenings, both HA-HA and HA-NA groups showed higher body mass indexes compared to the NA-NA group. Additionally, at follow-up, the HA-HA and HA-NA groups exhibited higher blood pressure, a higher prevalence of hypertension, elevated serum triglycerides and insulin levels, and lower levels of HDL cholesterol compared to the NA-NA group. Even after adjusting for BMI, significant differences persisted in HDL cholesterol levels and hypertension prevalence among the groups (HA-HA: 53.8%, HA-NA: 53.1%, NA-NA: 14.3%, *p* < 0.01). However, calcium scores and the prevalence of coronary plaques on CT scans were similar across all groups. In conclusion, women with PCOS and hyperandrogenism during their reproductive years exhibited an unfavorable cardiometabolic profile during their post-reproductive years, even if they changed to a normoandrogenic status.

## 1. Introduction

With a reported prevalence as high as 20%, PCOS is the most common endocrine disorder in women of reproductive age [1]. Previous studies have shown that PCOS is associated with unfavorable cardiometabolic risk factors, including obesity, an increased prevalence of insulin resistance, type 2 diabetes mellitus, hypertension, dyslipidemia, and metabolic syndrome (MetS) [2,3,4,5]. Some studies also described an increased risk for subclinical atherosclerosis and endothelial dysfunction in young women with PCOS compared to controls, reflected in increased carotid intima-media thickness (CIMT) and Coronary Artery Calcification Scores (CACs) [6,7]. Therefore, the international PCOS guideline recommends that all women with PCOS be assessed for cardiovascular risk factors and cardiovascular disease (CVD) risk [8].

The majority of studies assessing the abovementioned cardiometabolic risk factors focused on women at the time of PCOS diagnosis during their reproductive years [2,3,4,5,6,9,10]. The number of studies investigating cardiometabolic risk during subsequent years is limited, despite its importance, knowing that cardiovascular risk increases with age. The few studies in postmenopausal women with PCOS have shown an increased prevalence of hypertension but did not report an increased prevalence of actual cardiovascular disease [11,12,13].

The fact that an unfavorable cardiometabolic profile in women with PCOS during their reproductive years does not result in increased CVD at a later age is intriguing. One of the proposed explanations is that some PCOS characteristics, such as irregular cycles and elevated androgen levels, ameliorate with advancing age [14,15,16]. A second explanation is that a later menopause with prolonged estrogen exposure, as observed in women with PCOS, could be protective against CVD [17,18]. However, there is also evidence that there is no cardiovascular benefit to using estrogen in postmenopausal years [17,19]. Another theory is that only women with specific PCOS phenotypes have an increased CVD risk, in which higher androgen levels seem to play a key role [3,20,21]. If that is the case, women with PCOS and normal androgen levels throughout life may have a similar CVD risk compared to women without PCOS. The debate continues with respect to the implementation of CVD screening programs for women with PCOS, and accordingly, it is important to understand which specific PCOS phenotypes are more likely to develop CVD.

To date, no studies have investigated the association between PCOS characteristics at reproductive age and cardiometabolic status later in life. Therefore, our aim was to compare the cardiometabolic profiles during post-reproductive years between three groups: (1) women with PCOS who were hyperandrogenic at reproductive and post-reproductive age; (2) women with PCOS who were hyperandrogenic at reproductive age but normoandrogenic at post-reproductive age; and (3) women with PCOS who were normoandrogenic at reproductive and post-reproductive age.

## 2. Materials and Methods

### 2.1. Study Design and Setting

The current analysis involves a follow-up study using data from a standardized endocrinological screening at the Erasmus University Medical Center Rotterdam (Erasmus MC). Women who underwent this initial screening, received the diagnosis of PCOS, and also underwent a cardiovascular follow-up screening (part of the POPCORn and CREw-IMAGO studies) were eligible for this study [22]. The POPCORn and CREw-IMAGO studies were both multicenter, cross-sectional studies in which women with PCOS, premature ovarian insufficiency, and a history of pre-eclampsia underwent cardiovascular risk assessment, including a CIMT assessment and a contrast and non-contrast coronary CT (CCT) at either the Erasmus MC or University Medical Center Utrecht. All women provided written informed consent, and these studies have been approved by the Medical Research Ethics Committee of the University Medical Center Utrecht, University of Utrecht (registered at http://www.clinicaltrials.gov/ (accessed on 1 May 2023), unique identifier: NCT02616510, and at https://trialsearch.who.int/ (accessed on 1 May 2023), unique identifier: NTR5531).

### 2.2. Study Population

All women included in this study visited the outpatient fertility clinic in one of the abovementioned hospitals and were diagnosed with PCOS upon the initial screening, as published previously [23]. The diagnosis of PCOS was based on the Rotterdam criteria, and other relevant disorders were excluded [24]. According to these criteria, at least two of the following three symptoms were present: ovulatory dysfunction, polycystic ovarian morphology, and clinical and/or biochemical hyperandrogenism. Ovulatory dysfunction was defined as amenorrhea (absence of menstrual bleeding > 182 days) or oligomenorrhea (menstrual cycle > 35 days or <8 cycles per year). Polycystic ovarian morphology was defined as 12 or more follicles in one or both ovaries (2–9 mm in diameter) and/or increased ovarian volume (>10 cm^3^) using an ultrasound at <8 MHz. Clinical hyperandrogenism was defined as a modified Ferriman-Gallwey score ≥ 5 [8,25]. Biochemical hyperandrogenism was defined as a Free Androgen Index (FAI) > 4.5 and/or total testosterone > 3.0 nmol/L. If testosterone was measured with liquid chromatography-tandem mass spectrometry, we used an FAI cutoff > 2.9 and/or total testosterone > 2.0 nmol/L. Women were considered hyperandrogenic if they were clinically hyperandrogenic, biochemically hyperandrogenic, or both. Women were excluded if their androgenic status was missing at the initial screening or follow-up screening. We divided the study population into the following three groups: (1) women who were hyperandrogenic at initial screening as well as at follow-up screening (HA-HA), (2) women who changed from hyperandrogenic to normoandrogenic (HA-NA), and (3) women who were normoandrogenic at initial screening as well as at follow-up screening (NA-NA). Changing from a normoandrogenic status to a hyperandrogenic status is an uncommon phenomenon. As we expected, very few women fell into this category; they were mentioned in the results but not included in the analyses.

### 2.3. Measurements

**Figure 1:**
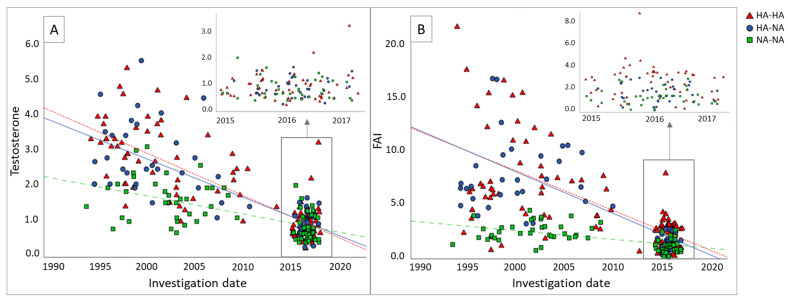
Testosterone levels (**A**) and FAI (**B**) of each participant at initial screening and follow-up screening. The red triangles represent women who were hyperandrogenic at initial screening and follow-up screening (HA-HA). The blue dots represent women who were hyperandrogenic at initial screening and normoandrogenic at follow-up screening (HA-NA). The green squares represent women who were normoandrogenic at both screenings (NA-NA). The initial screening took place on different dates, and the follow-up screening took place all around the same date. For testosterone, the slopes were −0.11 (HA-HA), −0.11 (HA-NA), and −0.05 (NA-NA). For FAI, the slopes were −0.27 (HA-HA), −0.34 (HA-NA), and −0.08 (NA-NA). The slopes for testosterone levels and FAI were significantly different for the HA-HA group compared to the NA-NA group (*p* < 0.01) and for the HA-NA group compared to the NA-NA group (*p* < 0.01). FAI, Free Androgen Index.

All included women underwent two assessments: an initial screening (standardized endocrinological screening at the time of diagnosis) and a follow-up screening (cardiovascular screening above the age of 45). Because the initial screening took place at different time points, the time interval between both screenings was different for every woman (Figure 1). Both screenings included a questionnaire, anthropometric measurements, hormonal evaluation, and transvaginal ultrasonography to assess ovarian volume and follicle count. The questionnaire included multiple questions about physical activity. In our study, physical activity was defined as all kinds of physical activities in hours per week, including walking, cycling, light housework, heavy housework, and all kinds of sports. For defining moderate exercise (to prevent CVD), we included walking, cycling, heavy housework, and all kinds of sports, and we excluded light housework, according to the American Heart Association [26]. At the follow-up screening, lipid levels (total cholesterol, high-density lipoprotein (HDL), low-density lipoprotein (LDL), and triglycerides), insulin, glucose, vitamin D, and brain natriuretic peptide (NT-proBNP), a marker for heart failure, were also assessed. Vitamin D deficiency was defined as a 25-OH-D serum level of <50 nmol/L, and FAI was calculated as (testosterone/SHBG)*100. Testosterone was first measured using 125I-radioimmunoassay (RIA) and later using LC-MS/MS. The intra-assay and inter-assay variation coefficients were <3% and <5%, respectively. Androstenedione was measured using LC-MS/MS. The intra-assay and inter-assay variation coefficients were <8% and <11%, respectively. SHBG was measured using the Immulite platform. The intra-assay and inter-assay variation coefficients were <4% and <5%, respectively. According to the National Cholesterol Education Program (NCEP) definition, MetS was diagnosed when ≥3 of the following features were present: waist circumference ≥ 88 cm, fasting glucose ≥ 6.1 mmol/L, blood pressure ≥ 130/85 mmHg, HDL < 1.3 mmol/L, and triglycerides ≥ 1.7 mmol/L. Dyslipidemia was defined as LDL ≥ 3.37 mmol/L, triglycerides ≥ 1.7 mmol/L, or the use of lipid-lowering medication. Diabetes was defined as the use of anti-diabetic medication or a self-reported diagnosis. Blood pressure was measured in the sitting position after at least five minutes of rest with a random-zero sphygmomanometer. Hypertension was defined as systolic blood pressure (SBP) ≥ 140 mmHg and/or diastolic blood pressure (DBP) ≥ 90 mmHg or the use of antihypertensive medication. The prevalence of CVD included myocardial infarction, stroke, and/or coronary heart disease.

CIMT was measured by ultrasound and defined as the distance between the lumen intima and the media-adventitia, measured three times on both sides. During the imaging part of the cardiovascular screening, a non-contrast-enhanced coronary CT (CCT) was performed to calculate the CACs using the Agatston scoring method [27], which was subsequently dichotomized as no calcium (CACs = 0 Agatston Units (AU)) or increased (CACs > 0 AU). Next, contrast-enhanced CCT angiography was performed to assess coronary artery atherosclerosis and stenosis. Coronary plaque was defined as having either calcified, non-calcified, or mixed plaque. Relevant coronary artery disease was defined as luminal stenosis of ≥50% or a CACs of ≥100 AU. The CCT protocol of this study has been published previously [22].

### 2.4. Statistical Analysis

Continuous variables are reported as medians with interquartile ranges (IQRs) and were compared between the three groups using the Kruskal-Wallis test and Mann-Whitney U test. Categorical variables are presented as numbers with percentages (%) and were compared between groups using the Chi-Square test. To adjust for multiple tests with pairwise testing, a Bonferroni correction was used. To adjust for BMI, linear and logistic regression analyses were used. Non-normally distributed variables were transformed to a natural logarithm prior to regression analysis. The initial screening took place at a different time for every patient. Therefore, the changes in testosterone levels and FAI between both screenings were converted into slopes to take differences in time intervals into account. Median slopes were compared between the three groups using the Kruskal-Wallis test. A *p*-value of <0.05 was considered statistically significant.

## 3. Results

### 3.1. Patient Characteristics at Initial Screening

A total of 130 women underwent both screenings and were therefore eligible for this study. Two of these women were excluded because their androgenic status was missing, and eight women were excluded from the analyses because they changed from a normoandrogenic status to a hyperandrogenic status. The HA-HA group consisted of 53 women, the HA-NA group of 32 women, and the NA-NA group of 35 women. Table 1 shows the baseline characteristics at initial screening for these three groups. The median age was similar in the three groups (32.3 years (HA-HA), 30.6 years (HA-NA), and 33.3 years (NA-NA), *p* = 0.14). Furthermore, no differences were observed in ethnicity (Northern European) or age at menarche between the groups. BMI at initial screening was significantly higher in the HA-HA group (27.5 kg/m^2^, IQR 23.1–32.4) and the HA-NA group (30.2 kg/m^2^, IQR 24.0–33.9) compared to the NA-NA group (22.4 kg/m^2^, IQR 20.6–25.5), *p* < 0.01. Waist circumference, hip circumference, and waist/hip ratio (WHR) were also significantly higher in the HA-HA group and the HA-NA group compared to the NA-NA group (all *p* < 0.01). Testosterone levels and the FAI were significantly higher in the HA-HA group and the HA-NA group compared to the NA-NA group (*p* < 0.01).

Figure 1 shows the testosterone levels (A) and the FAI (B) of each woman over time: at initial screening and at follow-up screening. The median time interval between both screenings was 15.8 years (IQR 12.1–19.5). For testosterone, the slopes were −0.11 (HA-HA), −0.11 (HA-NA), and −0.05 (NA-NA). This was significantly different between the HA-HA and NA-NA groups (*p* < 0.01) and between the HA-NA and NA-NA groups (*p* < 0.01). For FAI, the slopes were −0.27 (HA-HA), −0.34 (HA-NA), and −0.08 (NA-NA). This was also significantly different between the HA-HA group compared to the NA-NA group (*p* < 0.01) and the HA-NA group compared to the NA-NA group (*p* < 0.01).

### 3.2. Cardiometabolic Parameters at Follow-Up Screening

Table 2 shows the characteristics and cardiometabolic parameters at follow-up screening of the three groups. The median age of the women at follow-up screening was around 47 years and was similar in all groups. BMI was highest in the HA-HA group, followed by the HA-NA group, and both were significantly higher than the NA-NA group as a reference (30.1 kg/m^2^ (IQR 25.7–35.6), 30.1 kg/m^2^ (IQR 26.1–34.2), and 24.1 kg/m^2^ (IQR 22.1–28.7), respectively). The median change in BMI (delta BMI) from initial screening to follow-up screening was not different between the three groups. The number of women who lost weight compared to the initial screening was higher in the HA-NA group compared to the HA-HA group and NA-NA group (*n* = 9 (31%), *n* = 7 (13.5%), and *n* = 6 (17.6%), respectively); however, these differences were not statistically significant (*p* = 0.15). Including all kinds of physical activities (light housework, heavy housework, all kinds of sports, walking, and cycling) at the time of follow-up screening, we found similar durations of physical activities in the three groups (HA-HA: median of 22.8 h/week (IQR 13.9–30.0), HA-NA: median of 20.5 h/week (IQR 16.3–43.8), and NA-NA: 21.8 h/week (IQR 13.3–29.3), *p* = 0.79). In total, four women did not meet the recommended amount of moderate exercise, according to the American Heart Association. Systolic blood pressure was significantly different between both the HA-HA group (130.0 mm Hg (IQR 120.0–140.0)) and the HA-NA group (127.5 mm Hg (IQR 120.0–140.0)) compared to the NA-NA group (120.0 mm Hg (IQR 110.0–130.0)), *p* < 0.01. However, after adjusting for BMI, these differences were no longer statistically significant. The prevalence of hypertension was significantly higher in the HA-HA group compared to the NA-NA group (53.8 % vs. 14.3%, *p* < 0.01) and higher in the HA-NA group compared to the NA-NA group (53.1% vs. 14.3%, *p* < 0.01). These results remained statistically significant after adjusting for BMI and delta BMI. Serum triglycerides and the prevalence of metabolic syndrome were higher in the HA-HA and HA-NA groups compared to the NA-NA group, but these results did not remain significantly different after adjusting for BMI. Furthermore, HDL was lower in the HA-HA group compared to the NA-NA group, even after adjusting for BMI (1.4 mmol/L (IQR 1.1–1.6) vs. 1.82 mmol/L (IQR 1.52–2.32), *p* < 0.01). Insulin levels were significantly higher in the HA-HA group compared to the NA-NA group (92.0 pmol/L (IQR 52.0–161.0) vs. 66.0 pmol/L (IQR 45.0–95.0)), but did not remain statistically significant after adjusting for BMI. 

The mean CIMT and the prevalence of no CAC or any CAC were similar in all three groups (Table 3). Any form of coronary plaque on coronary CT angiography was present in 5 women (15.6%) in the HA-HA group, in none of the women in the HA-NA group, and in 3 women (16.7%) in the NA-NA group. This was not significantly different between the groups (*p* = 0.23). In the total study population, none of the women had relevant CVD.

## 4. Discussion

This study confirms that women with PCOS who are hyperandrogenic during their reproductive years have an unfavorable cardiometabolic profile during their post-reproductive years. Interestingly, we showed that this unfavorable cardiometabolic profile is independent of hyperandrogenic status during these post-menopausal years. In other words, women with PCOS who become normoandrogenic during these post-reproductive years still have an unfavorable cardiometabolic profile. This effect is partly BMI-driven, but the increased risk of hypertension and higher HDL levels were independent of BMI. We found no differences in markers for actual CVD, including mean CIMT, CACs, or the presence of any coronary plaque.

Our results support previous findings that in women with PCOS of reproductive age, an association exists between hyperandrogenism and an unfavorable cardiometabolic profile later in life [2,3,20,21,28]. The increased BMI, which also partially contributes to this profile, must also be taken into consideration as a serious cardiovascular risk factor, given that women with PCOS are more likely to be overweight or obese. However, we also showed that even though 31% of the women in the HA-NA group lost weight between the two screenings, this group still had a higher prevalence of hypertension and had lower HDL levels compared to the normoandrogenic women (even after adjustment for BMI and delta BMI). This is in line with our previous study by Daan et al., in which we reported a higher BMI, higher blood pressure, and higher lipid levels in the hyperandrogenic group compared to the normoandrogenic group [23]. The median age of those studied women was 27 years and 29 years, respectively. However, this association no longer existed when this was assessed in postmenopausal women with a mean age of 70 years [12]. Our results based on two time points (mean time interval 15.8 years) showed that, in women with PCOS whose hyperandrogenism disappeared with increasing age, the cardiovascular risk factors remained similar compared to the women with PCOS who were still hyperandrogenic during their post-reproductive years. This suggests that it may not be hyperandrogenism per se that exhibits a causal relationship with cardiovascular risk factors or cardiovascular disease. Dapas et al. demonstrated the complexity of PCOS and identified two major PCOS subtypes using unsupervised cluster analysis: a “reproductive” phenotype (driven by high LH and SHBG levels) and a “metabolic” phenotype (driven by high BMI, glucose, and insulin levels) [29]. In this hierarchical cluster analysis, the authors showed that testosterone levels did not discriminate between these two phenotypes, which reinforces the idea that it may not be hyperandrogenism per se that discriminates between PCOS subtypes but that insulin plays a more dominant role. Our study also showed that insulin levels were higher in the group of women who stayed hyperandrogenic and who changed from hyperandrogenic to normoandrogenic compared to women who were normoandrogenic at both assessments. This finding supports the results of earlier published studies [30,31,32,33]. One of these studies, including 1212 women with PCOS, showed that androgens decline with increasing age, with patients of 31–39 years old having less hyperandrogenemia compared to patients of 21–30 years of age, but that insulin resistance worsens [32].

Despite the unfavorable cardiometabolic profile in both groups of women who started hyperandrogenic, we only found a small number of women in our total study population with an increased CIMT, CACs, or the presence of any coronary plaque, which are predictors for actual cardiovascular events. On the one hand, this may reflect their relatively young age, since CVD events become more prevalent from age 60 and onward. On the other hand, there is still no evidence that women with PCOS suffer more often from CVD events compared to those without PCOS. A recently published study by Forslund et al. showed in a long-term follow-up study, including women with PCOS and controls with a mean age of 81 years, that all-cause mortality and CVD were similar in both groups [34]. This suggests that there is no increased risk for actual CVD events in women with PCOS compared to healthy controls without PCOS. This might be explained by the longer reproductive lifespan observed in women with PCOS, resulting in extended estrogen exposure, which is known to play a protective role against CVD [35,36]. It is also possible that these women have modified their risk factors through lifestyle adjustments and awareness gained after the initial screening. This was shown in the study by Brown et al., in which insulin resistance was significantly decreased in women with PCOS who had a follow-up visit after 0.5 to 3.9 years [14]. Nevertheless, in our study, in all three groups, the median BMI was higher at follow-up screening compared to initial screening, and the delta BMI was not significantly different between the groups. Lastly, it is also suggested that women with PCOS have better DNA repair and maintenance, which should give them a healthier cardiovascular profile compared to the general population [37]. If the latter is true, then there is still something to gain by investigating because they should actually have better prospects and therefore a lower incidence of CVD compared to controls.

Information about the use of oral contraceptives was available at the two time points, but information about the use of oral contraceptives and lifestyle adjustments between the two time points was not available and could be a possible confounder. Furthermore, the time intervals between both screenings were different, which could also have caused bias. However, adjusting for differences in time intervals did not change the results.

This study again emphasizes the importance of adequately educating women about a healthy lifestyle to prevent cardiometabolic risk factors, as well as the significance of early identification of insulin resistance, dyslipidemia, and hypertension. An important finding in our study is the discrepancy between the prevalence of hypertension during the cardiovascular screening and the prevalence of women who used antihypertensive medication. This indicates that some patients with hypertension are not receiving treatment and that there are also patients who were not screened regularly. According to the International Guideline, lifestyle intervention should be recommended in all those with PCOS and excess weight, and glycemic status should be assessed every one to three years in all women with PCOS [8]. Furthermore, GLP1-agonists appear to be promising in the treatment of metabolic problems in these patients [38,39].

In conclusion, women with PCOS and hyperandrogenism during their reproductive years had an unfavorable cardiometabolic profile during their post-reproductive years, even though androgens were normalized. This supports the idea that it is not hyperandrogenism per se that has a causal relationship with cardiovascular risk factors, and it highlights the key role of insulin in regulating these factors. Therefore, it is still important to adequately screen and monitor all women with PCOS throughout life and to perform long-term follow-up studies to determine the role of each factor in CVD to provide more definitive answers.

## Figures and Tables

**Table 1:** Characteristics of the three groups at initial screening.

	HA-HA (*n* = 53)	HA-NA (*n* = 32)	NA-NA (*n* = 35)	*p*-Value
Age (years)	32.3 (27.4–37.1)	30.6 (27.9–35.0)	33.3 (30.7–36.8)	0.14
NE ethnicity	42 (79.2)	30 (93.8)	30 (85.7)	0.19
Age at menarche (years)	13.0 (12.0–15.0)	13.0 (12.0–15.0)	13.0 (12.3–15.0)	0.72
Amenorrhoea	18 (34.0)	4 (12.9)	7 (20.0)	0.18
Oligomenorrhea	34.0 (64.2)	26 (83.9)	28 (80.0)	
Regular cycle	1 (1.9)	1 (3.2)	0 (0.0)	
BMI (kg/m^2^)	27.5 (23.1–32.3)	30.2 (24.0–33.9)	22.4 (20.6–25.5)	<0.01 ^1,2^
Waist (cm) *	94.0 (79.8–102.8)	96.0 (82.3–110.3)	75.0 (71.0–86.0)	<0.01 ^1,2^
Hip (cm) *	107.5 (98.0–114.5)	111.5 (100.8–121.0)	100.0 (89.0–109.0)	<0.01 ^2^
WHR	0.9 (0.8–0.9)	0.8 (0.8–1.0)	0.8 (0.7–0.8)	<0.05 ^1,2^
Testosterone (nmol/L)	2.79 (1.95–3.47)	2.48 (2.06–3.50)	1.64 (1.11–1.95)	<0.01 ^1,2^
FAI	6.7 (4.2–11.3)	6.4 (5.6–10.1)	2.5 (2.0–3.1)	<0.01 ^1,2^

HA-HA: women with PCOS who were hyperandrogenic at both screenings; HA-NA: women with PCOS who were hyperandrogenic at the initial screening and normoandrogenic at the follow-up screening; NA-NA: women with PCOS who were normoandrogenic at both screenings. Values are presented as medians (interquartile range) or as numbers (percentages). NE: Northern European; BMI: body mass index; WHR: waist/hip ratio; FAI: free androgen index. ^1^ Significant difference between the HA-HA and NA-NA groups after Bonferroni correction. ^2^ Significant difference between the HA-NA and NA-NA groups after Bonferroni correction. * Waist and hip circumference were missing for 13 women in the HA-HA group, 10 women in the HA-NA group, and 4 women in the NA-NA group.

**Table 2:** Cardiometabolic parameters of the three groups at follow-up screening.

	HA-HA (*n* = 53)	HA-NA (*n* = 32)	NA-NA (*n* = 35)	*p*-Value	Adjusted *p*-Value
Age (years)	46.9 (45.5–49.2)	47.4 (46.9–49.7)	47.6 (46.6–50.6)	0.20	0.51
BMI (kg/m^2^)	30.1 (25.7–35.6)	30.1 (26.1–34.2)	24.1 (22.1–28.7)	<0.01 ^2,3^	-
Delta BMI (kg/m^2^)	2.4 (0.7–5.5)	2.1 (−2.6–5.0)	2.0 (0.3–3.3)	0.80	-
Waist (cm) *	98.0 (88.5–109.0)	96.0 (84.0–107.0)	89.0 (80.3–96.3)	<0.05 ^2,3^	0.38
Hip (cm) *	101.0 (101.0–117.0)	112.0 (103.0–117.0)	104.5 (98.8–111.3)	0.18	0.45
Waist/Hip ratio *	0.9 (0.9–1.0)	0.9 (0.8–0.9)	0.8 (0.8–0.9)	<0.01 ^2^	0.25
Physical activity (hours per week) *	22.8 (13.9–30.0)	20.5 (16.3–43.8)	21.8 (13.3–29.3)	0.79	0.82
Systolic BP (mm Hg)	130.0 (120.0–140.0)	127.5 (120.0–140.0)	120.0 (110.0–130.0)	<0.01 ^2,3^	0.19
Diastolic BP (mm Hg)	85.0 (80.0–95.0)	80.0 (75.0–90.0)	80.0 (70.0–85.0)	<0.05 ^2^	0.16
Hypertension	28 (53.8)	17 (53.1)	5 (14.3)	<0.01 ^2,3^	<0.05 ^2,3^
Antihypertensive medication	12 (23.5)	5 (15.6)	1 (2.9)	<0.05 ^2^	0.07
Prevalent CVD	1 (1.9)	1 (3.1)	1 (2.9)	0.93	0.67
Lipid lowering medication	4 (7.5)	3 (9.4)	0 (0.0)	0.20	0.30
Total cholesterol (mmol/L) *	5.2 (4.3–5.9)	5.40 (4.2–6.2)	5.2 (4.6–5.8)	0.76	0.64
HDL cholesterol (mmol/L) *	1.4 (1.1–1.6)	1.5 (1.2–1.7)	1.82 (1.52–2.3)	<0.01 ^2,3^	<0.01 ^2^
LDL cholesterol (mmol/L) *	3.2 (2.6–4.1)	3.7 (2.5–4.2)	2.99 (2.7–3.6)	0.60	0.729
Triglycerides (mmol/L) *	1.0 (0.8–1.9)	1.1 (0.9–1.5)	0.85 (0.6–1.1)	<0.01 ^2,3^	0.18
Dyslipidemia *	31 (60.8)	21 (65.6)	13 (38.2)	0.05	0.16
Androstenedione (nmol/L)	2.9 (2.1–4.2)	2.5 (2.0–3.0)	2.6 (1.8–3.6)	0.13	0.02 ^2^
Testosterone (nmol/L)	1.1 (0.7–1.3)	0.7 (0.5–1.0)	0.7 (0.6–1.0)	<0.01 ^1,2^	<0.01 ^1,2^
FAI	2.7 (1.4–3.2)	1.8 (0.9–2.3)	1.2 (0.8–1.5)	<0.01 ^1,2^	<0.01 ^1,2^
NT-pro-BNP (>15 pmol/L)	5.0 (3.0–9.0)	6.0 (3.0–12.0)	6.0 (4.0–8.5)	0.81	0.72
Insulin (pmol/L)	92.0 (52.0–161.0)	95.0 (46.0–146.0)	66.0 (45.0–95.0)	<0.05 ^2^	0.34
Glucose (mmol/L)	5.5 (5.1–5.9)	5.1 (4.8–5.6)	5.30 (4.9–5.5)	0.13	0.47
Diabetes	7 (13.2)	2 (6.3)	1 (2.9)	0.20	0.24
Metabolic syndrome (NCEP definition) *	16 (37.2)	10 (35.7)	3 (8.6)	<0.05 ^2,3^	0.12

HA-HA: women who were hyperandrogenic at initial screening and at follow-up screening; HA-NA: women who were hyperandrogenic at initial screening and normoandrogenic at follow-up screening; NA-NA: women with PCOS who were normoandrogenic at initial screening and at follow-up screening. Values are presented as medians (interquartile range) or as numbers (percentages). The adjusted *p*-value was adjusted for BMI. BMI: body mass index; Delta BMI: difference in BMI between initial screening and follow-up screening; BP: blood pressure; HDL: high-density lipoprotein; LDL: low-density protein; FAI: free androgen index. ^1^ Significant difference between the HA-HA and HA-NA groups after Bonferroni correction. ^2^ Significant difference between the HA-HA and NA-NA groups after Bonferroni correction. ^3^ Significant difference between the HA-NA and NA-NA groups after Bonferroni correction. * Waist and hip circumference were missing for 11 women (*n* = 6 in the HA-HA group, *n* = 3 in the HA-NA group, and *n* = 2 in the NA-NA group). Physical activity was missing for 23 patients. Lipid levels were missing for 3 women (n = 2 in the HA-HA group and *n* = 1 in the NA-NA group). Metabolic syndrome was missing for 16 women (*n* = 10 in the HA-HA group, *n* = 4 in the HA-NA group, and *n* = 2 in the NA-NA group).

**Table 3:** Radiological findings of the three groups at follow-up screening.

	HA-HA	HA-NA	NA-NA	*p*-Value	Adjusted *p*-Value
Mean CIMT (um)	*n* = 47 0.6 (0.6–0.7)	*n* = 28 0.6 (0.6–0.7)	*n* = 33 0.6 (0.6–0.7)	0.93	0.82
CACs	*n* = 32	*n* = 18	*n* = 18	0.32	0.19
No CAC (0 AU)	25 (78.1)	15 (83.3)	17 (94.4)		
Any CAC (0–400 AU)	7 (21.9)	3 (16.7)	1 (5.6)		
Presence of coronary plaque	*n* = 31 5 (15.6)	0 (0.0)	*n* = 18 3 (16.7)	0.23	0.94
Relevant CVD	0 (0.0)	0 (0.0)	0 (0.0)	-	-

HA-HA: women who were hyperandrogenic at initial screening and at follow-up screening; HA-NA: women who were hyperandrogenic at initial screening and normoandrogenic at follow-up screening; NA-NA: women with PCOS who were normoandrogenic at initial screening and at follow-up screening. Values are presented as medians (interquartile range) or as numbers (percentages). The adjusted *p*-value was adjusted for BMI. CIMT: carotid intima-media thickness; CACs: coronary artery calcification score; AUs: Agatston Units; and CVD: cardiovascular disease. Relevant CVD was defined as luminal stenosis of ≥50% or a CACs of ≥100 AU.

## Data Availability

The data underlying this article will be shared on reasonable request by the corresponding author.
